# Designing for emergency remote blended and online education: a response to Bennett et al. (2017)

**DOI:** 10.1007/s11423-020-09892-0

**Published:** 2020-11-20

**Authors:** Cornelia Connolly, Tony Hall

**Affiliations:** School of Education, National University of Ireland, Galway, Ireland

**Keywords:** Educational design, Tertiary education, Online learning

## Abstract

This paper is in response to the article entitled “The process of designing for learning: understanding university teachers’ design work” (Bennett et al. in Educ Technol Res Dev 65(1):125–145, 2017). Design constitutes a fundamental part of what teachers do (Goodyear in HERDSA Rev Higher Educ 2:27–50, 2015). However, it has received negligible attention in the research literature. Bennett et al. make a significant contribution to knowledge by identifying and illustrating how university teachers engage in educational design. In particular, the paper identifies key areas for further support and the professional development of university teachers, including in the use of systematic design models and tools. This will help university teachers significantly, especially during the current pandemic has increased the design workload of university teachers as they endeavour to migrate and transtion their teaching online. Our response discusses Bennett et al. (2017) in the context of emergency remote teaching and the wholesale shift to new modalities of blended and online education. We also offer future suggestions arising from our review, including the importance of further international research on the topic.

## Introduction

Based on evaluation of the design processes of 30 teachers from 16 universities in Australia, the paper by Bennett et al. (2017) makes a significant contribution to our understanding of educational innovation by identifying and illustrating how university teachers’ undertake educational design. In this time of a global pandemic, when there has been a large-scale pivot to online education internationally, the paper provides useful insights for teachers’ design work in a time of crisis and the *shift to digital* at the tertiary level.

The paper and review of literature illustrate how design work is characterised in different countries and cultures, and how university teachers’ design work is generally focused in one of two ways: student-centred or teacher-centred. The authors report how, even within faculties where there is a strong collaborative approach, university teachers generally seem to have considerable autonomy in the decisions they make regarding educational design. Therefore, how can we better support university teachers as educational designers (a question that has gained renewed importance due to the current, global pandemic)? The paper provides important foundations for this conversation around how we can address this challenge.

## Impact and value

As outlined by the authors, teachers’ design work is a relatively new and emerging field of inquiry in educational research, and important questions remain unanswered, especially in relation to university teachers’ professional learning in educational design (Goodyear 2015). The effective professional development of teachers in educational designers, who play a crucial brokerage role in optimising technological innovations in complex educational settings, is at present neither well understood, nor supported effectively. McKenney and Schunn (2018, p. 1089) highlight how “[educational] designers are more powerfully positioned…to reach the vast majority of teachers and students experiencing curricula, assessments and professional development. But to date, limited research has been undertaken to support the performance of [educational] designers.” Significant advances have been made regarding the potential of technological innovation (Burden et al. 2019) and for learning (Wang and Hannafin 2005), however the training of educational designers who can maximise the educational benefits of using these new technologies has been largely and problematically overlooked. A corollary of this is the gap in the literature, and in our understanding, of university teachers as educational designers, and how they approach design as a foundational part of their work.

## Application

The paper by Bennett et al. (2017) casts new light on how university teachers engage in designing for their teaching. A first, really useful aspect of this study is its broad representativeness, engaging as it does with a diverse cross-section of university teachers, drawn from a range of different academic disciplines, and who, furthermore, teach in a variety of modalities: face-to-face, blended, online, etc.

Interestingly, despite the disciplinary diversity of respondents, and the nuances in their respective approaches, the paper reports common, shared practices across the varied cohort. A further, notable output of the research in this context is a descriptive model illustrating university teachers’ design processes, and the commonalities in the experience of university teachers as educational designers. This model will assist university educators who require heuristics to evaluate the impact of their designs for teaching on the learning experience of their students. Based on the 3P model (presage–process–product) (Biggs 1993) and Approaches to Teaching framework (Prosser and Trigwell 1997), the conceptual framing outlined in the paper provides a robust underpinning for understanding the interactions and influences that characterise university teachers’ design work. Their research-inspired model of teachers’ design processes is especially salient in the current situation and should be immediately useful for those now creating units of learning anew and or modifying existing courses.

## Limitations and constraints

The paper highlights the lack of bespoke resources and tools for teachers and university educators to use to enhance their design work. Notably, while generic design training may be useful, the authors note how teachers may need specially tailored approaches that are both relevant and responsive to the particular exigencies and requirements they face in designing/redesigning units of learning. The study is also solely focused on the Australian university sector, and it will be useful to see what emerges as the authors conduct similar research in other countries. This will enhance our understanding of how design is enacted by university teachers internationally, potentially widening the scope and reach of the research.

## Future suggestions

In terms of future developments, there are really interesting insights to be gleaned in respect of the characteristics of design work at tertiary level (Puente et al. 2013; Strange and Banning 2001). In particular, the authors identify key areas where university educators require support and professional development, to engage in systematic design of learning, in particular the use of design models and tools. The paper suggests that by making their design work more explicit—through the development and use of conceptual tools and design models—university teachers may be better able to navigate the tension between top-down (teacher-oriented) and bottom-up (student-centred) processes of design. This may furthermore help university teachers to better reconcile the needs of students with those of curriculum. Although the authors note how it is too early to be *prescriptive*, perhaps an area where the paper is lacking is in suggesting some specific approaches to explore how we can make university educators’ design work more explicit and systematic, e.g. conjecture maps (Sandoval 2014).

In the short timeframe available to university teachers to design new online learning experiences and, or redesign and update their existing classes amidst the Covid-19 pandemic, Bennett et al. (2017) makes for timely and informative reading. In the shift to digital enforced by emergency remote teaching, there is a need to raise our awareness around salient issues related to how university teachers see themselves as educational designers, and the commonalities of practices that we can learn from. Bennett et al. (2017) provide a useful, replicable descriptive model and important directions for enhancing the design work of educators in the academe, and therewith students’ learning experiences.
